# Antioxidant MMCC ameliorates catch-up growth related metabolic dysfunction

**DOI:** 10.18632/oncotarget.21965

**Published:** 2017-10-23

**Authors:** Liping Ju, Wenxin Tong, Miaoyan Qiu, Weili Shen, Jichao Sun, Sheng Zheng, Ying Chen, Wentao Liu, Jingyan Tian

**Affiliations:** ^1^ Shanghai Clinical Center for Endocrine and Metabolic Diseases, Shanghai Institute of Endocrine and Metabolic Diseases, Department of Endocrinology and Metabolism, Ruijin Hospital, Shanghai Jiao Tong University School of Medicine, Shanghai, China; ^2^ Department of Diabetes Complications and Metabolism, Beckman Research Institute of City of Hope, Duarte, CA, USA; ^3^ Shanghai Institute of Hypertension, Ruijin Hospital, Shanghai Jiao Tong University School of Medicine, Shanghai, China; ^4^ Laboratory of Endocrine and Metabolic Diseases, Institute of Health Sciences, Shanghai Institutes for Biological Sciences, Chinese Academy of Sciences, and Shanghai Jiao Tong University School of Medicine, Shanghai, China; ^5^ Key Laboratory of Shanghai Gastric Neoplasms, Department of Surgery, Ruijin Hospital, Shanghai Jiao Tong University, Shanghai Institute of Digestive Surgery, Shanghai, China

**Keywords:** catch-up growth, MMCC (MitoQuinone mesylate beta cyclodextrin complex), mitochondria, metabolic dysfunction, skeletal functions

## Abstract

Postnatal catch-up growth may be related to reduce mitochondrial content and oxidation capacity in skeletal muscle. The aim of this study is to explore the effect and mechanism of antioxidant MitoQuinone mesylate beta cyclodextrin complex (MMCC) ameliorates catch-up growth related metabolic disorders. Catch-up growth mice were created by restricting maternal food intake during the last week of gestation and providing high fat diet after weaning. Low birthweight mice and normal birthweight controls were randomly subjected to normal fat diet, high fat diet and high fat diet with MMCC drinking from the 4th week. MMCC treatment for 21 weeks slowed down the catch up growth and ameliorated catch-up growth related obesity, glucose intolerance and insulin resistance. MMCC administration significantly inhibited the peroxidation of the membrane lipid and up-regulated the antioxidant enzymes Catalase and MnSOD. In addition, MMCC treatment effectively enhanced mitochondrial functions in skeletal muscle through the up-regulation of the ATP generation, and the promotion of mitochondrial replication and remodeling. To conclude, this study demonstrates that antioxidant MMCC effectively ameliorates catch-up growth related metabolic dysfunctions by increasing mitochondrial functions in skeletal muscle.

## INTRODUCTION

Abnormal nutrition and metabolism in earlier stages of development frequently trigger metabolic disorders in adulthood. Fetal growth restriction usually develops catch-up growth that significantly increases the incidence of metabolic disorders in adults [1–3]. Abundant clinical observations demonstrate that catch-up growth sets the individual on the path to type 2 diabetes, obesity and cardiovascular diseases in adulthood [4–8]. However, during a critical period of life, usually when tissues exhibit a higher plasticity, environmental changes could reset the catch-up growth [9]. Moreover, the mechanisms of catch-up growth are reported related to the diminished mitochondrial mass and skeletal muscle function [10, 11].

Mitochondria is the primary controllers of cellular metabolism and forms a reticular network within mammalian skeletal muscle [12]. The network is properly organized through the mitochondrial dynamic interplay of fusion, fission, autophagy and mitochondrial biogenesis [13, 14]. The dynamic balance of mitochondria plays a critical role in the metabolism by regulating nutrients metabolism, producing ATP and generating heat [15]. MitoQuinone is one of the most extensively studied mitochondria-targeted antioxidants, which has been reported *in vitro* settings of mitochondrial oxidative stress and animal models of cardio-metabolic pathologies, such as ischemia/reperfusion, sepsis, and diabetes. Human studies demonstrated that it can be formulated into safety and effective pharmaceutical in parkinson’s disease and chronic hepatitis C virus disease [16, 17]. However, MitoQuinone may not be optimal for drug delivery in drinking water [18].

**Figure 1:**
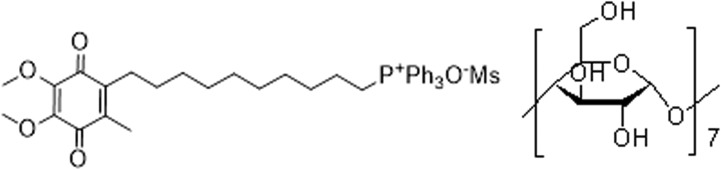
The constitutional formula of MMCC

Beta cyclodextrin, as well as mesylate, is widely used in food and medication producing, as it can enhanced the biocompatibility, water solubility and appetite without changing the active ingredient [19–21]. Hence, we adopted a water-soluble MitoQuinone mesylate beta cyclodextrin complex (MMCC) (Figure 1), to explore that whether long-term MMCC drinking could ameliorate catch-up growth related metabolic dysfunctions and the possible mechanism.

## RESULTS

### MMCC treatment ameliorate catch-up growth related metabolic dysfunction

**Figure 2:**
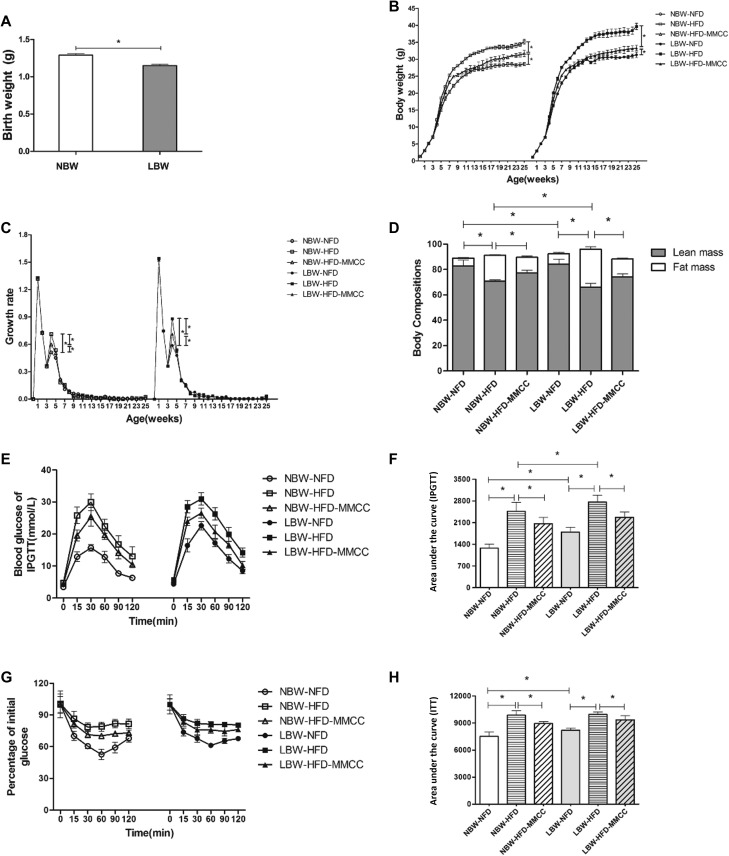
Growth phenotypes of the mouse model (**A**) Birth weight. (**B**) Body weight (1–25 w). (**C**) Growth rate (1–25 w). (**D**) Body composition in the 23th week. (**E**) Glucose levels during an intraperitoneal glucose tolerance test (2 g glucose/kg body weight) and Glucose AUC during the glucose tolerance test. (**F**) Insulin tolerance test (0.75 U insulin/kg body weight) and Glucose AUC during the insulin tolerance test, glucose levels were represented as percentage of initial glucose. Data represent mean ± SEM, *n* ≥ 4 mice/group. ^*^ *p* < 0.05.

Restricting maternal food intake during the last week of gestation resulted in a steady significant low body weight (1.15 ± 0.02 g, *p* < 0.05, Figure 2A), with no significant differences in number of pups and pregnant period (data not shown). Endurance administration of MMCC drinking from the 4th week slowed down the catch up growth, marked by significantly repressed growth rate at the 4th week and reduced body weight in adulthood (Figure 2B–2C).

In addition, Echo/MRI was used to analyze the body composition. Compared with normal fat diets feeding (NFD) groups, there is a significant increase of fat mass% in high fat diets feeding (HFD) groups, especially in low birth weight with high fat diets feeding (LBW-HFD) group (29.98 ± 1.99%, *p* < 0.05). However, MMCC drinking effectively ameliorated this trend and demonstrated to be sufficient in reducing catch-up growth related obesity (Figure 2D).

Further, intraperitoneal glucose tolerance test (IPGTT) and insulin tolerance test (ITT) were conducted to measure the physiology, metabolism and hormonal function in this mouse model. MMCC treatment remarkably ameliorate abnormal glucose intolerance and insulin resistance in LBW groups, especially in the HFD induced group (Figure 2E–2F).

### MMCC treatment enhanced mitochondrial oxidative balance

**Figure 3:**
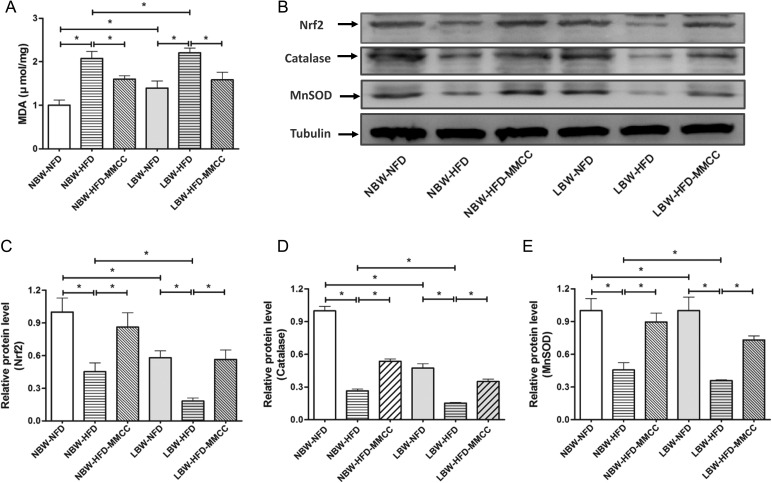
MMCC administration enhanced mitochondrial oxidative balance in gastrocnemius muscles The gastrocnemius muscles were rapidly extracted from the mice model and used to perform the following test. (**A**) MDA levels. (**B**–**E**) Western blot images and quantitative band density analyses of endogenous antioxidants: Nrf2, Catalase, and MnSOD. Data represent mean ± SEM, ^*^ *p* < 0.05

Mitochondrial oxidative balance in skeletal muscle was investigated in this study. The muscular malondialdehyde (MDA) level, sign of peroxidation of the membrane lipid, was examined by a MDA Assay Kit, which demonstrated a significant elevated trend in LBW-HFD mice (Figure 3A), while partially reduced by MMCC treatment.

In addition, we analyze the protein levels of the antioxidant system, such as Nrf2 (a redox-sensitive, basic leucine zipper protein that regulates the transcription of several antioxidant genes) and antioxidant enzymes Catalase and MnSOD (Figure 3B). Corresponding, the remarkable reduced levels of Nrf2, Catalase and MnSOD in LBW-HFD group were reversed by endurance MMCC administration (Figure 3C–3E).

### MMCC promoted mitochondrial replication and remodeling

**Figure 4:**
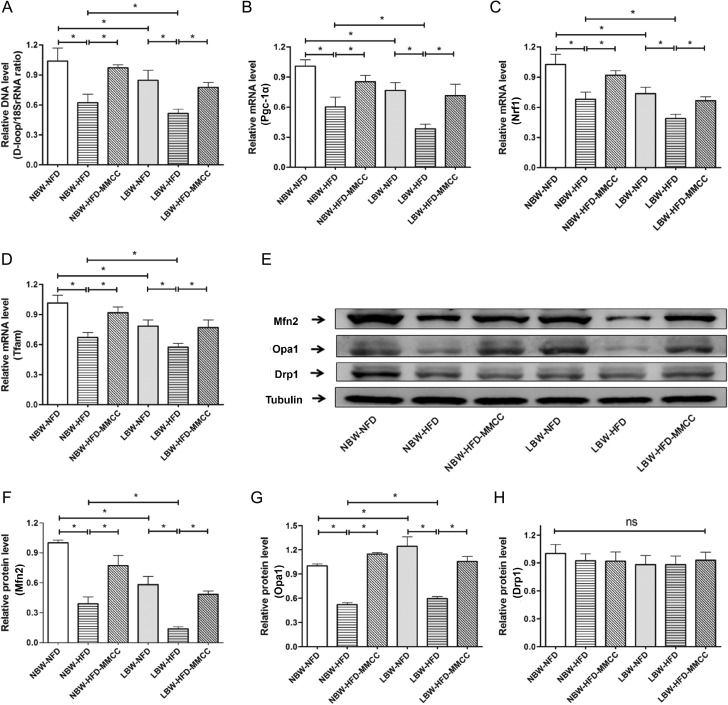
MMCC promoted mitochondrial replication and remodeling in gastrocnemius muscles The gastrocnemius muscles were rapidly extracted from the mice model and used to perform the following test. (**A**) mtDNA levels. Q-PCR analysis of the mitochondrial biogenesis-related genes: (**B**) Pgc-1α, (**C**) Nrf1 and (**D**) Tfam. (**E**–**H**) Western blot images and quantitative band density analyses of mitochondrial dynamic remodeling: Mfn2, Opa1 and Drp1. Data represent mean ± SEM,^*^ *p* < 0.05.

As was shown in Figure 4A, the mitochondrial DNA copy number, acted as a marker for mitochondrial content, was notable reduced in LBW mice, especially after HFD inducement. MMCC treatment significantly increased the mitochondrial DNA copy numbers, which indicated the up-regulation of mitochondria replication.

Consistently, regulators of mtDNA transcription and replication, such as peroxidase body growth-activated receptor gamma coactivator 1 (Pgc-1α), the transcription factor nuclear respiratory factor 1 (Nrf1) and mitochondrial transcription factor A (Tfam) were down-regulated in LBW-HFD and NBW-HFD mice, while were significantly reversed by MMCC administration (Figure 4B–4D).

As mitochondrial dynamic remodeling is a considerable part of mitochondrial function, we further investigated the levels of mitochondrial fission and fusion related proteins: dynamic-related protein-1 (Drp1), mitofusion-2 (Mfn2) and opticatropy-1 (Opa1) (Figure 4E). Western bolt results showed that LBW mice developed mitochondrial remodeling dysfunction while MMCC treatment effectively enhanced it, marked by up-regulated Mfn2 and Opa1 expression (Figure 4F–4G). However, mitochondrial fission-related protein Drp1 was not significantly changed between groups (Figure 4H).

### MMCC up-regulated the β-oxidation of fatty acid and ATP generation in gastrocnemius muscles

**Figure 5:**
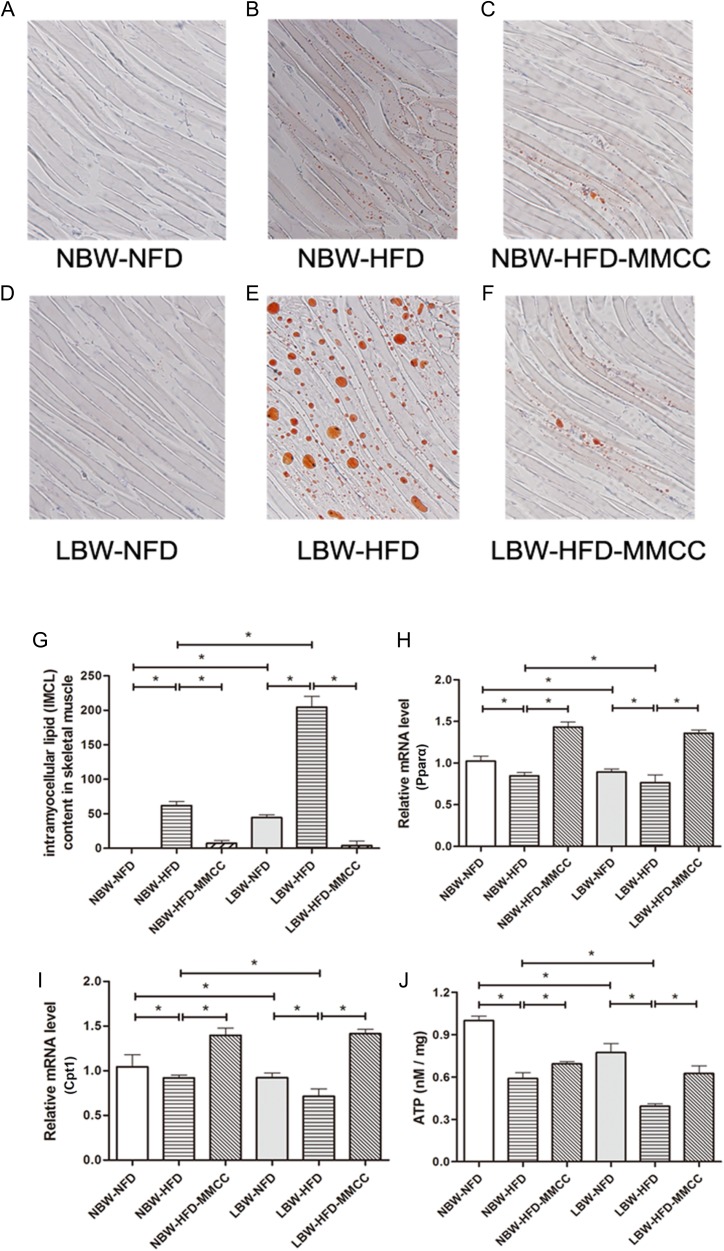
MMCC up-regulated the β-oxidation of fatty acid and ATP generation in gastrocnemius muscles Intramyocellular lipid content was measured by Oil Red O staining. Observation under light microscope (100 × original magnification) : (**A**) NBW-NFD, (**B**) NBW-HFD, (**C**) NBW-HFD-MMCC, (**D**) LBW-NFD, (**E**) LBW-HFD, (**F**) LBW-HFD-MMCC. (**G**) Quantitative analysis of gastrocnemius muscles sections after Oil red O staining. Q-PCR analysis of β-oxidation of fatty acid related genes in skeletal muscle: (**H**) Pparα, (**I**) Cpt1. (**J**) ATP levels of all groups in skeletal muscle. Data are represent mean ± SEM, *n* ≥ 4 mice/group. ^*^p < 0.05, ^**^p < 0.01.

Oil Red O staining was conducted to estimate the intramyocellular lipid content in skeletal muscle. Observation under light microscope (100 × original magnification) indicated that there was hardly intramyocellular lipid deposition in NFD groups, while was indistinct in NBW-HFD and evident in LBW-HFD (Figure 5A–5G). Remarkably, MMCC treatment reduced the lipid content in both NBW-HFD and LBW-HFD groups.

Further, mRNA levels of peroxisome proliferator-activated receptors alpha (Pparα) and carnitine palmitoyltransferase 1 (Cpt1) were tested to investigate the β-oxidation of fatty acid in skeletal muscle. As expected, endurance MMCC drinking effectively invert the decreasing trend of mRNA levels of Pparα and Cpt1 in LBW mice (Figure 5H–5I).

Besides, to estimate the mitochondrial function, an ATP Assay Kit was used to test the ATP levels, which revealed obvious reductions of ATP levels in both NBW-HFD (0.59 ± 0.04 nM/mg, *p* < 0.05) and LBW-HFD (0.39 ± 0.02 nM/mg, *p* < 0.05) mice, while MMCC drinking significantly increased the ATP levels (Figure 5J).

## DISCUSSION

Fetal origins of adult diseases theory indicates fetal growth restriction usually followed by type 2 diabetes, obesity and cardiovascular diseases in adulthood. And the detrimental effects of low birth weight are often amplified by rapid postnatal catch-up growth [4–8]. Catch-up growth during a critical period of life could be reset by environmental changes [9]. A mouse model study [22] indicates two distinct growth spurts in rodents. Growth rate of the 4th week had the strongest correlation with body weight and glucose tolerance at the age of 6 months, and was demonstrated to be the most critical growth phase for the development of obesity in a C57BL/6J mouse model [23]. Therefore, we chose the 4th week as our intervention time to testify whether MMCC drinking could ameliorate catch-up growth related metabolic dysfunctions.

As it had been reported diminished skeletal muscle mitochondrial mass and function were related with catch up growth [10, 11]. Interventions of increasing the skeletal muscle mitochondrial mass and function could be potential effective methods to limit catch up growth, even ameliorate catch up growth related adult metabolic disorders. It has been reviewed that both regular endurance exercise [13] and antioxidant therapy [15] effectively reduced diseases through positive effects on mitochondrial biogenesis and function. We established an endurance exercises mouse model and found that long-term exercise training from the 4th week is sufficient to ameliorate catch-up growth and related metabolic disturbances in the 25th week by promoting mitochondrial functions in skeletal muscle [23]. In this study, we aimed to determine whether antioxidant could ameliorate catch-up growth, and explored the possible underlying mechanisms.

Studies of mitochondria-targeted antioxidant are emerging and growing vigorously, and the best characterized mitochondria-targeted antioxidant to date is MitoQuinone, which consists of a ubiquinone moiety linked to a TPP by a ten-carbon alkyl chain. Within mitochondria, it is absorbed to the matrix-facing surface of the inner mitochondrial membrane. However, pure MitoQuinone is a tar-like or waxy semi-solid and very hard to transfer between containers. Hence, we adopted MMCC, a water-soluble MitoQuinone mesylate beta cyclodextrin complex, which enhanced the solubility while remained the activity of MitoQuinone.

Postnatal catch-up growth developed serious obesity and glucose intolerance, as well as diminished mitochondrial content and mitochondrial dysfunction at the 25th week were demonstrated in this study. However, we found that postnatal catch up growth and related metabolic disorders was ameliorated through long term MMCC drinking after weaning. The benefit consequences had been seen in LBW-HFD-MMCC, as well as NBW-HFD-MMCC, including adiposity reduction, glucose tolerance amelioration, and enhancement of mitochondrial function. Furthermore, MDA level in skeletal muscle was effectively reduced and the expression of endogenous antioxidant was increased. At the same time, intramyocellular lipid of skeletal muscle showed the greatest increased in catch up growth mice, as well as weakened β-oxidation of fatty acid. In our research, MMCC drinking could ameliorate oxidative stress, increase antioxidant enzymes, reduce the intramyocellular lipid and promote β-oxidation of fatty acid in skeletal muscle.

The adaptability of skeletal muscle is also associated with mitochondrial morphological plasticity, and mitochondria are dynamic and readily adapt to changes in cellular energy demands through network remodeling and continuous fusion and fission [24]. Previous research have found reduced levels of fusion proteins Mfn2 and Opa1, but no alterations in fission proteins Drp1 or Fis1 in skeletal muscle in type 2 diabetic individuals [25]. While another study found Mfn1 and Mfn2 but not Opa1 were decreased, and Drp1 and Fis1 were increased in skeletal muscle of HFD-induced obesity mice [26]. However, we demonstrated Mfn2 and Opa1 were decreased in HFD groups, especially in LBW-HFD (catch up growth mice), while Drp1 were not significantly changed among all groups. These differences among the researches may be caused by different experimental design and animal models. In our study, MMCC drinking could effectively invert the decreased mitochondrial fusion protein Mfn2 and Opa1 which induced by catch-up growth.

In summary, our data demonstrate that long-term antioxidant MMCC drinking beginning at the 4th week of age prevented rapid postnatal catch-up growth and exhibited beneficial systematic metabolism effects through increased mitochondrial content and skeletal muscle function. Targeting mitochondrial pharmacology has emerged as an effectively therapeutic method for catch-up growth related metabolic dysfunction.

## MATERIALS AND METHODS

### Mouse model

20 Six-to-eight-week-old virgin female C57BL/6J mice were caged with 10 eight-to-ten-week-old C57BL/6J males (SLAC Laboratory Animal, Shanghai, China) in a temperature-controlled room with a 12:12-h light-dark cycle. Low birthweight mice were resulted by restricting maternal food intake during the last week of gestation [27]. In this study, 18 males from 5 litters in normal birth weight (NBW) groups and 18 males picked from 15 litters in low birth weight (LBW) groups were further randomly distributed into three additional experimental groups after weaning: (1) normal fat diet (NFD); (2) high fat diet (HFD, 60% kcal for fat, Research Diets, D12492); and (3) high fat diet with MMCC drinking. We designated these six groups as NBW-NFD (*n* = 6), NBW-HFD (*n* = 6), NBW-HFD- MMCC (*n* = 6), LBW-NFD (*n* = 6), LBW-HFD (*n* = 6), LBW-HFD- MMCC (*n* = 6).

All procedures were approved by the Institutional Animal Care (IACUC #10-09011), and all experiments involving animals were conducted in conformance with the principles of laboratory animal care (NIH Publication, 8th edition, 2011).

### Drinking protocol

After weaning, mice in MMCC groups were administered 500 μM MMCC in their drinking water ad libitum, with fresh MMCC solutions given twice a week. Mice of other groups were supplied with water alone.

### Body composition and growth rate

Animal Body Composition Analyzer (EchoMRI-100, Houston, TX, USA) were used to assess fat mass and lean mass.

Body weight was recorded weekly from birth to 24 weeks. The growth rate was calculated as:

$$GR=\left(W_{n}-W_{n-1}\right)/W_{n-1}$$

where *W* is body weight and *n* is the age in weeks [22].

### IPGTT and ITT

All mice were overnight fasting (12–14 h) by removal to a clean cage without food and weighted before measurement in week 24 (IPGTT) and in week 25 (ITT), respectively. Blood was collected from the tail vein immediately before injection (0 min) and at 15, 30, 60, and 120 min after injection and measured using the glucose analyser Ascensia Elite (Bayer HealthCare AG, Leverkusen, Germany).

### Animal sacrifice and oil red O staining

All of the mice were overnight fasted and sacrificed with an intraperitoneal injection of 10% chloral hydrate (100 μl/20 g body weight).The gastrocnemius muscles of the left posterior limb, subcutaneous adipose tissue, epididymis gastrocnemius muscles adipose tissue and brown adipose tissue were rapidly extracted, weighted, frozen in liquid nitrogen and stored at −80°C. Furthermore, gastrocnemius muscles of the right posterior limb were rapidly extracted and fixed in 4% paraformaldehyde to make frozen sections and oil red O staining as soon as possible. Then, the slices were going through several process: drying for 10 minutes, incubation in 75% alcohol for 10 seconds, incubation in oil red O for 15 minutes in dark, differentiation by 75% alcohol for 2 seconds, water wash for 1 minutes, re-staining by hematoxylin and finally mounting by glycerol gelatin.

### MDA (malondialdehyde) and ATP (adenosine triphosphate) assay

MDA and ATP levels in skeletal muscle were measured using a Lipid Peroxidation MDA Assay Kit (Beyotime, Shanghai, China) and an ATP Assay Kit (Beyotime, Shanghai, China), respectively. The ATP assay kit is based on the principal that luciferase firefly requires ATP to provide energy for the development of fluorescence. When the luciferase firefly and fluorescein are excessive, in a certain concentration range, the fluorescence is proportional to the concentration of ATP. Both measurements followed the manufacturer’s instructions. Protein concentrations in samples were determined using a BCA assay kit (Thermo Scientific, USA). MDA and ATP equivalents are expressed as nmol/mg tissue protein and normalized to NBW-NFD levels.

### Western blot

30 mg gastrocnemius muscles were lysed by adding 500 μl RIPA and ground 3 times at 60HZ for 30 seconds using the homogenizer (ARTMICR, USA), while total proteins of each group were obtained from the supernatant after centrifugation at 12000g for 20 minutes (4°C). Soluble lysates (20 μg/lane) were resolved on SDS polyacrylamide slab gels (NuPAGE 10% Bis-Tris; Invitrogen, Carlsbad, CA, USA). After electrophoresis, proteins were blotted onto a PDVF membrane and blocked with 5% (w/v) nonfat milk/ Tris-buffered saline Tween 20 (TBST) for 1 h at room temperature. Membranes were incubated overnight at 4°C with primary antibodies directed against β-tubulin (1:3000), Nrf2 (1:1000), MnSOD (1:20000), catalase (1:3000), Mfn2 (1:1000), Opa1 (1:1000), and Drp1 (1:1000) in 5% BSA/TBST (w/v). After washed three times with TBST and membranes were then incubated with an HRP-linked secondary antibody for 1 h at room temperature. Electro-chemoluminescence (ImageQuant LAS4000, USA) were used to develop Western blot images. Antibodies against β-tubulin were purchased from Cell Signaling Technology (Danvers, MA, USA). Antibodies against Nrf2, MnSOD, catalase, Mfn2, Opa1 and Drp1, were purchased from Santa Cruz Biotechnology (Santa Cruz, CA, USA).

### Total DNA/RNA isolation and real-time PCR

Total DNA was extracted from 25 mg of tissue using the QIAamp DNA Mini kit, while total RNA was extracted from 25 mg of tissue using TRIzol reagent (Invitrogen, USA). Samples of 2.0 μg total RNA were reverse transcribed into cDNA. Quantitative real-time PCR was performed using a Light Cycler 480 Real Time PCR System (USA) with SYBR Premix EX Taq TM (TaKaRa, Dalian, China). The mouse 18S ribosomal RNA (rRNA) gene served as the endogenous reference gene. A melting curve was performed to ensure specific amplification. Relative differences in PCR products among the groups were assessed using the 2^-∆∆CT^ method. The results are presented as fold differences of the NBW-NFD. All the cycling conditions were as follow: hold at 95°C for 30 s, amplification at 95°C for 5 s and 60°C 30 s for 40 cycles, then dissociation at 95°C for 15 s, 60°C for 30 s and 95°C for 15 s. Related gene sequences are followed as:

Nrf1: forward: 5′-CGGAAACGGCCTCATGTGT-3′; reverse:5′-CGCGTCGTGTACTCATCCAA-3′. Tfam: forward: 5′-GGAATGTGGAGCGTGCTAAAA-3′; reverse:5′-ACAAGACTGATAGACGAGGGG-3′. Pgc-1α:forward: 5′-AGCCGTGACCACTGACAACGAG-3′; reverse: 5′-GCTGCATGGTTCTGAGTGCTAAG-3′.Mitochondrial D-loopforward: 5′-AATCTACCATCCTCCGTG-3′; reverse: 5′-GACTAATGATTCTTCACCGT-3′. 18SrRNA: forward:5′-CATTCGAACGTCTGCCCTATC-3′; reverse: 5′-CCTGCTGCCTTCCTTGGA-3′. Pparα: forward:5′-TCGGCGAACTATTCGGCTG-3′; reverse 5′-GCACTTGTGAAAACGGCAGT-3′. Cpt1: forward: 5′- TCTTCTTCCGACAAACCCTGA-3′; reverse 5′- GAGACGGACACAGATAGCCC-3′.

### Statistical analysis

The results are expressed as mean ± SEM. Group comparisons of other data were performed using multivariable ANOVA, with MMCC drinking as a covariate and followed by post hoc least significant difference tests. Statistical analyses for Body weight, Growth rate, IPGTT and ITT were performed using a repeated measure ANOVA. All data were analyzed with the using SPSS for Windows Version 17.0. *p* values < 0.05 were considered significant.
